# Auditory Processing Disorders in Elderly Persons vs. Linguistic and Emotional Prosody

**DOI:** 10.3390/ijerph18126427

**Published:** 2021-06-14

**Authors:** Anna Rasmus, Aleksandra Błachnio

**Affiliations:** Institute of Psychology, Kazimierz Wielki University in Bydgoszcz, 85-064 Bydgoszcz, Poland; alblach@ukw.edu.pl

**Keywords:** central auditory process, emotional prosody, linguistic prosody

## Abstract

Background: Language communication, which is one of the basic forms of building and maintaining interpersonal relationships, deteriorates in elder age. One of the probable causes is a decline in auditory functioning, including auditory central processing. The aim of the present study is to evaluate the profile of central auditory processing disorders in the elderly as well as the relationship between these disorders and the perception of emotional and linguistic prosody. Methods: The Right Hemisphere Language Battery (RHLB-PL), and the Brain-Boy Universal Professional (BUP) were used. Results: There are statistically significant relationships between emotional prosody and: spatial hearing (*r*(18) = 0.46, *p* = 0.04); the time of the reaction (*r*(18) = 0.49, *p* = 0.03); recognizing the frequency pattern (*r*(18) = 0.49, *p* = 0.03 (4); and recognizing the duration pattern (*r*(18) = 0.45, *p* = 0.05. There are statistically significant correlations between linguistic prosody and: pitch discrimination (*r*(18) = 0.5, *p* = 0.02); recognition of the frequency pattern (*r*(18) = 0.55, *p* = 0.01); recognition of the temporal pattern; and emotional prosody (*r*(18) = 0.58, *p* = 0.01). Conclusions: The analysis of the disturbed components of auditory central processing among the tested samples showed a reduction in the functions related to frequency differentiation, the recognition of the temporal pattern, the process of discriminating between important sounds, and the speed of reaction. De-automation of the basic functions of auditory central processing, which we observe in older age, lowers the perception of both emotional and linguistic prosody, thus reducing the quality of communication in older people.

## 1. Introduction

In the aging society, persons in late adulthood have become the object of increasing interest. Thus, it is difficult to define a single model of old age. The changes that happen during this period and the level of psychological functioning of the elderly are currently being investigated. The gathered results often indicate a decline in skills and competencies, which determine the cognitive impairment, activity limitations, and quality of life of the elderly [1,2,3,4,5,6]. Among other things, the aging process depends on one’s sex, education, health status, intellectual and physical activity, lifestyle, economic situation, and personality traits [7]. Accordingly, various compensation methods, and even various functional therapies, are proposed to increase mental performance.

This paper discusses auditory processing in elderly persons. Although auditory processing is regularly investigated, the results are often limited to early developmental stages, such as childhood. The current state of knowledge on how changes in auditory processing affect individuals in late adulthood is not satisfactory. The literature suggests that this process may intensify with age [8,9]. The deterioration central-level functions leads to verbal communication problems, both in terms of making oneself understandable and understanding others.

According to the experts at the National Institute on Ageing, sensory or perception changes might be early biomarkers of dementia [10,11]. It is important to identify cognitive deficits as early as possible in order to delay or reduce the risk of developing dementia, such as Alzheimer’s disease.

Moreover, the experts at the National Institute on Aging claim that focusing on problems with sensory and perception processing may potentially be a way to delay or prevent dementia [10]. Therefore, it is important to better understand auditory processing in elderly persons, both among the cognitively normal elderly and among individuals with expected mild cognitive impairment (MCI).

Poor results, in terms of respective auditory processing indicators, may be an early symptom of dementia; This is why it is important to thoroughly analyze these processes in elderly persons, so that deterioration processes can be stopped with the use of modern technologies.

A striking feature of normal aging is the significant frugality in understanding spoken language—despite its high requirements in terms of the speed of processing, the sharpness of hearing, and the working memory capacity, all of which become more or less reduced in normal aging [12,13,14].

### 1.1. Central Auditory Processing

The hearing process takes place on a number of levels; it begins the moment a sound reaches the ear, and it continues until the sound has been fully perceived. Hearing can be divided into peripheral and central types. Peripheral hearing is dependent on the correct construction and functioning of the peripheral auditory system and neural pathways that deliver information to the auditory cortex [15,16].

The American Speech–Language–Hearing Association (ASHA) defines auditory processing as the “perceptual (neural) processing of auditory information in the central nervous system and neurobiological activity that gives rise to electrophysiologic auditory potentials” [17].

The available sources discuss the phenomenon of auditory processing and auditory processing disorders mainly within the context of children and youth; there is limited data concerning the processes and difficulties that it might cause in elderly persons.

Well-functioning central processing requires many functions in different parts of the brain, such as sound location and lateralization, auditory discrimination, the identification of the properties of auditory patterns, the temporal processing of sounds, the ability to discern an acoustic signal from a jamming signal, and the ability to recognize a distorted acoustic signal [18]. Research shows that many elderly persons have problems in all the areas of auditory processing, and that these problems intensify with age and are associated with social isolation, depression, and a deteriorated quality of life.

Recently, researchers and clinicians have focused their activities around the central auditory process, as the limitations of the audiogram have been identified [9,17]. An audiogram provides information about sensitivity. According to the classic reports of Ettore Bocca, patients with significant central auditory dysfunction may have normal audiograms and may also reproduce normal audiometric thresholds.

There is still a lack of standardized tools available to test the central auditory process. One of the few tools available is the Brain-Boy Universal Professional (BUP), which is part of the Warnke method. This method is based on the automation of auditory, visual, and motor perceptual processing. For this method, the basic prerequisites for learning perceptual processing must be well-developed, and processing speed must be improved and automated. The advantage of this method is that it is based on the assumption that higher-level language functions depend on the correct functioning of low-level functions. Low-level functions are understood as basic cognitive functions—which include, for example, the extraction of basic acoustic features, such as the frequency or duration of a sound.

The phenomenon of individuals experiencing discomfort in their hearing despite obtaining a normal audiometric test recording is referred to as “hidden hearing loss”. This describes disorders that involve more temporal aspects of hearing, thus limiting the ability to understand speech that is degraded by noise, reverberation, speed, limited articulation, or the localization of sound sources.

### 1.2. Linguistic and Affective Prosody

The term prosody refers to the non-linguistic aspects of speech that are important for the communication process. These include intonation, speech rate, pauses, rhythmicity, accent, and melody. Linguistic prosody refers to stressing the right syllables in a word or words in a sentence as well as the intonation relevant to the type of utterance (e.g., a statement, question, negation, or imperative). Affective prosody reflects the emotions of the speaker.

The ability to make correct use of prosodic information is very important for understanding the intentions and psychological condition of the interlocutor. Affective prosody strengthens or weakens the linguistic message. If the two are contradictory, the prosodic information is more reliable to the recipient than the linguistic message. Affective prosody probably engages the perisylvian area in the right hemisphere (the same as linguistic functions engage the areas in the left hemisphere). The area in the left hemisphere of the brain (which is analogous to Broca’s area) is responsible for the accurate perception of affective prosody, and the region of the right hemisphere of the brain (which is analogous to Wernicke’s area) is responsible for the comparisons [19].

The current literature describes the significant decline in central auditory processing among older adults. Further, the speech prosody (both emotional and linguistic) may deteriorate with age [11,20,21,22]. A significant body of research on central auditory processing during the aging period implements self-description questionnaires. The present study adds to these findings by measuring low-level auditory functions through the use of the Brain-Boy Universal Professional (BUP). The present study sought to determine whether the reduction in individual basic auditory functions correlate with the level of linguistic and emotional prosody. Two research questions were asked:

1. What is the correlation between the auditory low-level functions and the emotional prosody among the group of elderly people?
2. What is the correlation between the auditory low-level functions and the linguistic prosody among the elderly group?

## 2. Materials and Methods

The following research tools were used in this study:

The Right Hemisphere Language Battery (RHLB-PL) was used to examine the language and communication functions of the right hemisphere. This test examines the understanding of the emotional intonation of nonsensical sentences.

- The emotional prosody test from the Polish version of the Right Hemisphere Language Battery (RHLB-PL) taps into one’s ability to understand the emotional intonation of meaningless sentences. There are 16 recorded utterances preceded by 3 examples. The participant chooses the card with the name of the emotion (e.g., joy, sadness, or anger) that is relevant to the heard sentence. Respondents may also provide a verbal answer [23].
- The linguistic prosody test from the Right Hemisphere Language Battery taps into the ability to understand the sentence modes of meaningless utterances. In the linguistic prosody test, there are also 16 recorded utterances preceded by 3 examples. The respondent chooses the card indicating one of the three possible sentence modes (i.e., question, negation, or statement). Respondents may also provide a verbal answer.

The Brain-Boy Universal Professional (BUP) device is a professional device functioning based on scientifically standardized data, which enables the therapist to obtain valid and true information when testing low-level central functions. The device has high technical parameters. In particular, it offers features that are specially designed for each low-level function, such as the volume-adjustment of earphones from 72 dB to 110 dB; show/do not show visual confirmation; the repetition of the last signal/task; the choice of signal-mode, click or noise-burst; the logarithmic approximation determination of the approximate order threshold with a max. of 40 signals; successive approximation for the quick determination of the current order threshold, thus avoiding effects of fatigue; and randomized order of and intervals between user-defined maximums and minimums.

The Brain-Boy Universal Professional (BUP) can be used for the testing and training of the following low-level functions: visual and auditory order thresholds, spatial hearing, pitch discrimination, visual and auditory motor-timing, choice reaction time, frequency pattern recognition, and recognition of the duration of tones.

The efficiency of this training tool has been proven through a scientific study carried out by the Medical University of Hanover. In this way, the BUP is a professional device, working on the basis of scientifically standardized data, that enables the therapist to obtain valid and true information when testing basic central functions. The low level functions tested by Brain-Boy Universal Professional are presented in Table 1.

The research group consisted of 20 persons aged M = 63.4 (SD = 5.21) years (min = 60; max = 79). There were 13 male and 7 female respondents. Inclusion criteria were the lack of dementia or MCI. Hearing sensitivity was evaluated using a Maico MA-1 screening audiometer (with 4 frequencies (0.5, 1, 2, 4 kHz) and a sound intensity range from 15 to 50–5 dB increments) with DD-45 audiometric earphones that were equipped with external noise attenuators. Only subjects whose hearing threshold did not exceed 20 dBHL in tonal audiometry qualified for participation in the study. WHO assumes that 20 dBHL is the threshold for normal hearing.

Statistical analyses were completed using the STATISTICA 13.1 package. To perform correlation analyses, we used the Pearson coefficient. The analyses were performed assuming a level of statistical significance of *p* = 0.05

## 3. Results

The emotional prosody score was low; the mean sten score was 4. The mean sten score for linguistic prosody was 6. The obtained results are presented in Table 2.

The mean values obtained for the auditory low-level functions are shown in Table 3.

In order to identify correlations between the results of the respective Brain-Boy tests and the results of the emotional prosody test, Pearson’s *r* coefficients were used. The obtained results are presented in Table 4.

There were no statistically significant relationships between emotional prosody and auditory order threshold, pitch discrimination, or auditory motor coordination.

There was a statistically significant relationship between spatial hearing and emotional prosody (*r*(18) = −0.46, *p* = 0.04). The results suggest that the relationship between spatial hearing and emotional prosody is negative and moderate. There was a statistically significant relationship between emotional prosody and the time of the reaction (*r*(18) = −0.49, *p* = 0.03). This result suggests that there exists a moderate, negative correlation between the time of the auditory reaction and emotional prosody. Respondents who scored lower on the analyzed low-level functions needed more time to complete the task. The relationship between recognizing the frequency pattern and emotional prosody (*r*(18) = −0.49, *p* = 0.03) was negative and moderate; The relationship between recognizing the duration pattern and emotional prosody (*r*(18) = −0.45, *p* = 0.05) was moderate. Respondents who scored lower on the analyzed low-level functions needed a longer stimulus.

In order to determine the relationships between the results achieved in the respective Brain-Boy tests and in the linguistic prosody test, Pearson’s *r* coefficients were used. The obtained results are presented in Table 5.

There were no statistically significant relationships between linguistic prosody and auditory order threshold, directional hearing, or auditory motor coordination. There was a statistically significant correlation between linguistic prosody and pitch discrimination (*r*(18) = −0.5, *p* = 0.02).

The correlation between linguistic prosody and the reaction time was also significant (*r*(18) = −0.57, *p* = 0.01). This result suggests that there exists a negative, moderate relationship between the time of the auditory reaction and emotional prosody. Respondents who scored lower on the analyzed low-level functions needed more time to complete the task. The relationship between recognizing the frequency pattern and linguistic prosody (*r*(18) = −0.55, *p* = 0.01) was also significant and moderate. The significant correlation between recognizing the temporal pattern and emotional prosody (*r*(18) = −0.58, *p* = 0.01) was moderate. Respondents who scored lower on the analyzed low-level functions needed a longer stimulus.

## 4. Discussion

In the respondent group of older adults without symptoms of dementia, disorders were more frequent in the perception of emotional prosody than in the perception of linguistic prosody. This supports the substantial evidence present in the literature [22], which suggests that the understanding of affective prosody decreases with age. Older adults are less capable of differentiating the levels of emotional intensity than are younger adults. Both the left and right hemisphere are responsible for prosodic processes. Nevertheless, the right hemisphere seems to specialize in the emotional communication that is expressed through prosodic channels, gestures, and the face, while the left hemisphere processes the temporal structure of speech.

Our results suggest that persons who achieved worse results for spatial hearing made more errors in their interpretation of utterances in terms of emotional prosody. Further, as a result of having slower reactions to auditory stimuli, participants had more difficulties with the proper interpretation of utterances during the emotional prosody test. We observed that persons’ problems with recognizing and identifying differences in the pitch of sounds, and problems with recognizing and identifying differences in the length of sounds, resulted in the misinterpretation of the emotional intonation of the utterances.

Participants who found it difficult to discriminate between minimum differences in the pitch of sounds also had more problems with identifying the mode of the utterances. Persons who found it difficult to recognize and identify differences in the length of sounds had more problems with interpreting the linguistic intonation of the utterances.

Older adults with better performances on the reaction time and selection tests also had more difficulty with evaluating the utterances on the linguistic prosody test.

### 4.1. Temporal Aspects of Processing vs. Emotional and Linguistic Prosody

The present results prove the relationship between the temporal aspects of processing and prosody. Processing auditory stimuli in a specific order affects such properties as rhythm and stress. The available literature [24] suggests that the temporal arrangement affects the understanding of the interlocutor’s intentions (e.g., whether they ask a question or make an exclamation; i.e., the mode of the sentences in the linguistic prosody test), as well as the understanding of the meaning of utterances in terms of the emotions conveyed. Temporal processing also determines the ability to distinguish between similar words. Our results of the temporal pattern recognition test are significantly related both to the results of the emotional prosody test and the linguistic prosody test; in both cases, the relationship is moderate. This means that persons who had difficulties with identifying differences in the lengths of sounds also had more problems with interpreting emotional and linguistic intonation. Meanwhile, the test that evaluated the auditory stimuli order threshold did not reveal any statistically significant relationships with the level of utterance performance, in terms of interpreting the emotional prosody or the linguistic prosody of utterances. This is probably due to the fact that the Brain-Boy auditory test evaluates the amount of time needed to recognize two stimuli that are emitted one after another, and to determine which stimulus occurred first. Accordingly, the auditory order threshold could correlate with the recognition of smaller sections of the language, such as syllables or phonemes, which is associated with examining the phonemic hearing and is beyond the scope of this paper.

Temporal processing of information by the central nervous system, and its relationships with prosody, may be related to other cognitive functions. The utilized tests do not evaluate one single isolated function; rather, both the BUP tests and the emotional and linguistic prosody tests engage the attention and memory functions—in this case, the working memory. Research confirms that all of the cognitive functions can be analyzed in temporal segmentation categories. Such aspects of cognitive functioning as memory, attention, language, movement, and emotional evaluation are embedded in a single time matrix [25]. In Baddeley’s working memory model—in the short-term memory pool—information is not only stored, but also processed, in a controlled manner. With auditory processing, the functioning of the phonological loop, subordinated to the central executive system in the working memory, is important. The phonological loop stores information in the acoustic code and refreshes it, by means of the articulatory loop, through voiceless repetition. Accordingly, the working memory may process new auditory stimuli, making use of long-term memory resources—which enables, for example, the interpretation of the emotional or linguistic prosody of utterances, as discussed in this paper. The literature highlights a relationship between auditory processing disorders and short-term auditory memory. Verbal memory is engaged in the storage and processing of information associated with speech, but it is also responsible for the pace of the articulation of words [11,26].

### 4.2. Tone Differentiation vs. Linguistic and Affectional Prosody

For the tests that examined tone differentiation and frequency pattern recognition, created using the Brain-Boy Universal Professional (BUP) device, the respondents performed below the reference values. Tone differentiation is associated with the prosody [27,28]; accordingly, problems with tone differentiation lead to further problems with word and sentence stress, which may disrupt the interpretation process as well as the process of making one’s own utterances. Research suggests that even a simple sentence may be interpreted in a number of different ways through shifting the sentence stress from one word to another [27,29,30]. Sentence stress is associated with minor pitch changes. The fact that the respondents’ results were significantly below the reference values suggests that they have problems noting the subtle pitch differences, which results in the misinterpretation of the utterances they hear. Aside from this, they also have problems with stressing a sentence appropriately and with changing the pitch of their voice to reflect their intentions; as a result, those who hear their message may interpret it in the wrong way. Moreover, the research suggests [15,27,31] that the ability to differentiate tones translates to the ability to distinguish between pairs of vowels: e–i, o–u, as the difference between them results from tone frequency. Thus, it could be concluded that tone differentiation is important not only for interpreting an utterance but also for understanding specific words that differ, for example, by one vowel only.

In the case of some words, this may entirely change the meaning of a word and, consequently, the meaning of the message. Tone differentiation should also be associated with the affective aspects of prosody. The significantly lower result achieved in the respondent group may also translate to problems with understanding and conveying emotions in utterances, which is discussed in detail below.

The present results suggest statistically significant relationships between the above-mentioned tests and prosody, both in terms of tone differentiation and frequency pattern recognition. This relationship is moderate. This means that persons who have problems with tone differentiation also have problems with recognizing the mode of utterances (the linguistic prosody test examined the ability to recognize affirmative, interrogative, and imperative modes). These results confirm former theoretical deliberations [22,27]. Meanwhile, considering the results of the emotional prosody test, there exist statistically significant moderate relationships between frequency pattern recognition and the interpretation of the emotional prosody of utterances. In practice, this means that persons who find it difficult to recognize and identify differences in the pitch of sounds also have problems with interpreting emotional intonation; thus, these persons have problems identifying whether an utterance addressed to them was supposed to be joyful or sad. No such differences were found between the tone differentiation test and the utterance interpretation in the emotional prosody test. The theory suggests, however, that there exists a link between the ability to differentiate between tones with the affective perception of utterances. It is stressed that there exists an important link between recognizing minimum differences in pitch and effective prosody in making and receiving utterances [27,31]. This suggests that the evaluation of emotional prosody is affected by a number of other factors that are not discussed in this paper.

The aging process is associated with the deterioration of the right hemisphere [32,33,34]. These changes influence the range of spoken language comprehension processes. As a result of this deterioration, elderly persons become less sensitive to the pitch contour, which is more effectively processed by the right hemisphere. Another current area of interest is in audiological changes associated with age that may reduce the effectiveness of the temporal and frequency resolutions. Such changes may suggest a selective impairment of sensitivity to time or pitch changes. Reduced performance of any of these factors may be concealed by the compensatory use of coexisting prosodic features. The obtained results focus on a more targeted and effective rehabilitation protocol for seniors.

The present research has important limitations. As the aging process progresses, the peripheral hearing of seniors systematically deteriorates. It is estimated that hearing impairment in old age affects about 25% of people aged 65–74 years, and 60–70% of people over 75 years. The present sample was not randomly selected, but was dictated by the inclusion criterion which was possessing normal audiometric test results; the audiometric score could not exceed 20 dBHL. The collected research sample is small in size; therefore, the results cannot be considered representative of the heterogenous population of the elderly. Furthermore, we are aware that a limitation of the study is the use of screening audiometry without taking into account speech frequencies, which, in the Polish language, range between 800–8000 Hz.

## 5. Conclusions

The present study posed questions about the relationship between low-level auditory function and emotional and linguistic prosody, with results showing correlations between linguistic prosody and the selected low-level functions (i.e., speech discrimination, voice reaction time, the frequency pattern test, and the duration pattern test). On the other hand, in terms of emotional prosody, results showed statistically significant relationships with spatial hearing, choice reaction time, the frequency pattern test, and the duration pattern test.

## Figures and Tables

**Table 1:** Low-level function testing features offered by the Brain-Boy Universal Professional.

		Interval-Range
Auditory Order Threshold	Two tones are produced. The child hears two clicks through headphones—one click from each side—and they must figure out which click was the first one, while the level of difficulty is steadily increasing.	5–800 ms; sequence threshold—the amount of time between two auditory stimuli; it is necessary to arrange them in the correct order; the task lasts for 3 min or up to 3 errors in 7 consecutive trials.
Spatial Hearing	The direction of a click that seems to come from a point near the middle of the head must be identified.	20–800 µm; the task takes 40 trials or up to 3 errors in 7 consecutive trials.
Pitch Discrimination	Two sounds of different pitches are produced. The person practicing must recognize their difference and order.	92–1%; the task takes 40 trials or up to 3 errors in 7 consecutive trials.
Auditory Motor Coordination—Finger-Tapping	In this program, the user must press buttons according to clicks heard in the headphones that are coming alternately from two sides. Respondent must press two buttons in synchrony with the clicks. If it is completed correctly, the speed increases.	160–900 ms; delay time of pressing the buttons relative to the alternating sounds (left hand for the left ear, right hand for the right ear); the task lasts 80 s.
Choice-Reaction Time	It is a combination of pitch discrimination and reaction. Here, the user must identify the difference between the two tones given, know where they came from, and name them as quickly as possible. For instance, they must find out as quickly as possible which side the deeper tone was on.	value in ms; the time taken from hearing a stimulus to marking the lower stimulus button; there are 40 pairs of sounds presented in this task.
Frequency Pattern Test	This program produces three tones, one of which is different and must be recognized. In the case of success, the duration of the tones and the intervals in between are shortened.	10–800 ms; the decision of where a sound of a different pitch out of a sequence of three sounds is located; The measured duration of sounds needed to recognize the correct stimulus; the task takes 40 trials or up to 3 errors in 7 consecutive trials.
Duration Pattern Test	This program produces three tones, one of which is longer and must be recognized. In the case of success, the duration of the tones is shortened.	10–800 ms; the decision of where a sound is located, with a sequence of three sounds of varying length; the measured duration of the sounds (with increasing difficulty of the task, the duration of the sounds is gradually reduced) needed to recognize the correct stimulus; the task takes 40 trials or up to 3 errors in 7 consecutive trials.

**Table 2:** Emotional and linguistic prosody.

	Mean	Min	Max	SD
Emitional Prosody	10.15	7	14	2.46
Lingusitic Prosody	11.15	7	16	2.87

**Table 3:** Auditory low-level function and prosody tests.

	Mean	Min	Max	SD
Auditory Order Threshold	144.14 ms	24.00	400.0	119.28
Spatial Hearing	137.42 µm	22.00 µm	320.00 µm	104.59
Pitch Discrimination	25.11%	1.00%	40.00%	15.21
Auditory Motor Coordination	388.85 ms	152.00 ms	588.00 ms	144.66
Choice-Reaction Time	860.38 ms	522.00 ms	1970.00 ms	445.06
Frequency Pattern Test	212.00 ms	34.00 ms	450.00 ms	143.12
Duration Pattern Test	219.25 ms	16.00 ms	500.00 ms	167.71

**Table 4:** Auditory low-level function and emotional prosody.

Central Auditory Process	Emotional Prosody			
	*r*(X,Y)	*r* ^2^	*t*	*p*
Auditory Order Threshold	−0.27	0.08	−1.21	0.24
Spatial Hearing	−0.46	0.2	−2.18	0.04
Pitch Discrimination	−0.44	0.2	−2.06	0.06
Choice Reaction Time	−0.49	0.24	−2.37	0.03
Auditory Motor Coordination	−0.3	0.09	−1.3	0.21
Frequency Pattern Test	−0.49	0.24	−2.38	0.03
Duration Pattern Test	−0.45	0.2	−2.11	0.05

*r*, Pearson correlation coefficient; *r*^2^, coefficient of determination; *t*, *t*-test value; *p*, *p*-value.

**Table 5:** Auditory low-level function and linguistic prosody.

Central Auditory Process	Linguistic Prosody			
	*r*(X,Y)	*r* ^2^	*t*	*p*
Auditory Order Threshold	−0.40	0.16	−1.88	0.08
Spatial Hearing	−0.39	0.15	−1.77	0.09
Pitch Discrimination	−0.5	0.26	−2.49	0.02
Choice Reaction Time	−0.57	0.33	−2.97	0.01
Auditory Motor Coordination	−0.33	0.11	−1.47	0.16
Frequency Pattern Test	−0.55	0.3	−2.8	0.01
Duration Pattern Test	−0.58	0.34	−3.01	0.01

*r*, Pearson correlation coefficient; *r*^2^, coefficient of determination; *t*, *t*-test value; *p*, *p*-value.

## Data Availability

Not applicable.
